# A geodatabase of blood pressure level and the associated factors including lifestyle, nutritional, air pollution, and urban greenspace

**DOI:** 10.1186/s13104-021-05830-2

**Published:** 2021-11-18

**Authors:** Alireza Mohammadi, Elahe Pishgar, Neda Firouraghi, Nasser Bagheri, Ali Shamsoddini, Jaffar Abbas, Behzad Kiani

**Affiliations:** 1 Department of Geography and Urban Planning, Faculty of Social Sciences, University of Mohaghegh Ardabili, Ardabil, Iran; 2 Department of Human Geography, Faculty of Earth Science, Shahid Beheshti University, Tehran, Iran; 3 Department of Medical Informatics, School of Medicine, Mashhad University of Medical Sciences, Mashhad, Iran; 4 Visualization and Decision Analytics (VIDEA) Lab, Centre for Mental Health Research, Research School of Population Health, College of Health and Medicine, The Australian National University, Canberra, Australia; 5 The Australian Geospatial Health Lab, Health Research Institute, The University of Canberra, Canberra, Australia; 6 Department of Human Geography, Faculty of Humanities, Islamic Azad University, Marvdasht Branch, Marvdasht, Iran; 7 Antai College of Economics and Management, and School of Media and Communication, Shanghai Jiao Tong University, Shanghai, China

**Keywords:** Air pollution, Green space, Geographical information system, Hypertension, Blood pressure, Nutritional factors, Spatial analysis

## Abstract

**Objectives:**

Hypertension is a prevalent chronic disease globally. A multifaceted combination of risk factors is associated with hypertension. Scientific literature has shown the association among individual and environmental factors with hypertension, however, a comprehensive database including demographic, environmental, individual attributes and nutritional status has been rarely studied. Moreover, an integrated spatial-epidemiological approach has been scarcely researched. Therefore, this study aims to provide and describe a geodatabase including individual-based and socio-environmental data related to people living in the city of Mashhad, Iran in 2018.

**Data description:**

The database has been extracted from the PERSIAN Organizational Cohort study in Mashhad University of Medical Sciences. The data note includes three shapefiles and a help file. The shapefile format is a digital vector storage format for storing geometric location and associated attribute information. The first shapefile includes the data of population, air pollutants and amount of available green space for each census block of the city. The second shapefile consists of aggregated blood pressure data to the census blocks of the city. The third shapefile comprises the individual characteristics data (i.e., demographic, clinical, and lifestyle). Finally, the fourth file is a guide to the previous data files for users.

## Objective

Hypertension is one of the prevalent health problems that causes almost 10.4 million deaths worldwide [1] and known as a growing challenge in countries with ageing population [2]. It is estimated that more than three-quarters of hypertensive patients are from developing countries [3]. A recent study has shown that the hypertension prevalence is 25% in Iran between 2004 and 2018 [4], which varies across the country [5]. This geographical variation designates the importance of geospatial analyses. Hypertension causes some severe complications such as cardiovascular and kidney diseases [6], moreover, it is one of the risk factors for worse COVID-19 outcomes [7, 8]. Hypertension occurrence is associated with a combination of genetic and lifestyle-related factors such as lack of physical activity [9], smoking [10], obesity [9, 11], alcohol consumption [11] and environmental factors, including the amount of available green space and air pollution [12–20]. The relationship between air pollution factors (pm1, pm 2.5, pm10) and hypertension has been confirmed [12, 13, 21–24]. Targeted strategies should be applied to prevent and control hypertension [25]. However, spatial analyses of all mentioned risk factors are needed to provide evidence-based information to develop appropriate interventions in areas with high priority. Geospatial analysis that is conducted by the geographical information system (GIS) help researchers and policymakers to analyze, identify, and visualize geographical patterns of diseases [26–28]. Thus, in this study, we used GIS to link and quantify risk factors to describe and provide a geodatabase of the PERSIAN Organizational Cohort study [29], as a set of individual and socio-environmental factors, to determine the blood pressure level of people living in Mashhad City in 2018. This geodatabase is a practical tool to identify high-risk areas of hypertension and exploring socio-environmental factors in future studies for managing resources and implementing targeted interventions [30].

## Data description

In this study, 5938 samples were obtained through the PERSIAN Cohort study in Mashhad [29]. The city of Mashhad is the second most populous city in Iran, located in the northeast of the country [31]. The data have been linked to the census tract level that is the finest available geographical level in Iran [32]. Hypertension is the most significant risk factor for cardiovascular disease (32% of all deaths worldwide in 2019) [33]. It is also associated with severity and mortality in patients with coronavirus 2019 (COVID-19) [7, 8]. Therefore, the connection with the two main global causes of death conveys the importance of this database.

This data note includes three data files and a help file (Table 1). Data file 1 has population and air pollutants data that have been aggregated into the ‏2018 census tracts. This data file contains census tracts identification number, total population, male and female population, air quality index (AQI) and green space per capita. It also contains the amount of air pollutants in each census tract including sulfur dioxide (SO_2_), particulate matter less than 10 microns (PM_10_), carbon monoxide (CO), nitrogen dioxide (NO_2_), particulate matter less than 2.5 microns (PM_2.5_) and ozone (O_3_).

**Table 1:** Overview of data sets

Label	Name of data file	File types (file extension)	Data repository and identifier (DOI or accession number)
Data file 1	Population and air pollutants data	Polygonal features shapefile format (*.shp)	Harvard Dataverse https://doi.org/10.7910/DVN/GKJUSL [39]
Data file 2	Aggregated blood pressure data	Polygonal features shapefile format (*.shp)	Harvard Dataverse https://doi.org/10.7910/DVN/GKJUSL [39]
Data file 3	Individual data	Point features shapefile format (*.shp)	Harvard Dataverse https://doi.org/10.7910/DVN/GKJUSL [39]
Data file 4	Help file	MS Excel file (*.xlsx)	Harvard Dataverse https://doi.org/10.7910/DVN/GKJUSL [39]

Data file 2 includes aggregated blood pressure data into the census tracts containing number of individuals without hypertension, cases in the pre-hypertensive stage (Elevated), cases with type 1 and type 2 blood pressure [34] and finally number of total cases. According to the definition of hypertension [34], the participants were categorized into normal (systolic blood pressure (SBP) less than 120 mm Hg and diastolic blood pressure (DBP) less than 80 mm Hg), elevated (SBP between 120 and 129 mm Hg and DBP less than 80 mm Hg), stage 1 hypertension (SBP between 130 and 139 mm Hg or DBP between 80 and 89 mm Hg) and stage 2 hypertension (SBP more than 140 mm Hg or DBP more than 90 mm Hg).

Data file 3 includes the individual data of participants and their nutritional habits. The data include age, sex, SBP, DBP, lifestyle information (height, weight, body mass index, amount of meat and vegetable intake, alcohol intake, smoking and salt consumption).

Data file 4 is a guide in excel format to using data of data files 1–3. The data do not include any direct identification data of patients, in data file 3, the point locations have been jittered around a 500-m circle to protect the individuals’ privacy.

These data files can be used by researchers in different disciplines such as health geography, urban planning, medicine and healthcare ecosystem research. A multilevel regression model can be used to quantify the impact of area level characteristics on individuals’ hypertension [35]. GIS has a great capacity to integrate diverse data from different sources including spatial, temporal and descriptive components into one framework [36–38]. These data can help to investigate the potential relationship of lifestyle and environmental risk factors with hypertension. Table 1 shows the details of each dataset and provides access links to these data.

## Limitations

One of the limitations of the data is that the PERSIAN COHORT study may not generalizable to the total population in Mashhad. Further, we obtained the air pollution data by spatially interpolating the data of meteorological stations, which may not be 100% accurate due to the modifiable areal unit problem.

## Data Availability

The data described in this data note can be freely and openly accessed on Harvard Data-verse under the address of https://doi.org/10.7910/DVN/GKJUSL [39]. Please see Table 1 and the reference list for details and links to the data.

## Abbreviations

GIS: Geographical Information Systems
AQI: Air Quality Index
PM10: Particulate matter less than 10 microns
PM2.5: Particulate matter less than 2.5 microns
SO2: Sulfur dioxide
CO: Carbon monoxide
NO2: Nitrogen dioxide
O3: Ozone
SBP: Systolic blood pressure
DBP: Diastolic blood pressure

## References

[1] Stanaway JD, Afshin A, Gakidou E, Lim SS, Abate D, Abate KH (2018). Global, regional, and national comparative risk assessment of 84 behavioural, environmental and occupational, and metabolic risks or clusters of risks for 195 countries and territories, 1990–2017: a systematic analysis for the Global Burden of Disease Study 2017. Lancet.

[2] Park JB, Kario K, Wang J-G (2015). Systolic hypertension: an increasing clinical challenge in Asia. Hypertens Res.

[3] Yusufali AM, Khatib R, Islam S, Alhabib KF, Bahonar A, Swidan HM (2017). Prevalence, awareness, treatment and control of hypertension in four Middle East countries. J Hypertens.

[4] Oori MJ, Mohammadi F, Norozi K, Fallahi-Khoshknab M, Ebadi A, Gheshlagh RG (2019). Prevalence of HTN in Iran: meta-analysis of published studies in 2004–2018. Curr Hypertens Rev.

[5] Ebrahimi M, Heidari-Bakavoli AR, Mazidi M, Moohebati M, Azarpazhooh MR, Nematy M (2016). Prevalence of hypertension, pre-hypertension and undetected hypertension in Mashhad, Iran. Mediterr J Nutr Metab.

[6] Olsen MH, Angell SY, Asma S, Boutouyrie P, Burger D, Chirinos JA (2016). A call to action and a lifecourse strategy to address the global burden of raised blood pressure on current and future generations: the Lancet Commission on hypertension. Lancet.

[7] Chen RY, Jie G, Xubin D, Xiaohan Y, Yuanqi S, Yang H (2020). Influence of blood pressure control and application of renin-angiotensin-aldosterone system inhibitors on the outcomes in COVID-19 patients with hypertension. J Clin Hypertens.

[8] Huh K, Lee R, Wonjun J, Minsun K, Hwang IC, Dae HL, Jaehun J (2020). Impact of obesity, fasting plasma glucose level, blood pressure, and renal function on the severity of COVID-19: a matter of sexual dimorphism?. Diabetes Res Clin Pract.

[9] Li W, Wang D, Wu C, Shi O, Zhou Y, Lu Z (2017). The effect of body mass index and physical activity on hypertension among Chinese middle-aged and older population. Sci Rep.

[10] Talukder MH, Johnson WM, Varadharaj S, Lian J, Kearns PN, El-Mahdy MA (2011). Chronic cigarette smoking causes hypertension, increased oxidative stress, impaired NO bioavailability, endothelial dysfunction, and cardiac remodeling in mice. Am J Physiol Heart Circulatory Physiol.

[11] Singh S, Shankar R, Singh GP (2017). Prevalence and associated risk factors of hypertension: a cross-sectional study in urban Varanasi. Int J Hypertens.

[12] Yang B-Y, Guo Y, Bloom MS, Xiao X, Qian ZM, Liu E (2019). Ambient PM1 air pollution, blood pressure, and hypertension: insights from the 33 Communities Chinese Health Study. Environ Res.

[13] Zhang Z, Dong B, Li S, Chen G, Yang Z, Dong Y (2019). Exposure to ambient particulate matter air pollution, blood pressure and hypertension in children and adolescents: a national cross-sectional study in China. Environ Int.

[14] Yu Y, Yao S, Dong H, Wang L, Wang C, Ji X (2019). Association between short-term exposure to particulate matter air pollution and cause-specific mortality in Changzhou, China. Environ Res.

[15] Chen T, Lang W, Li X (2020). Exploring the impact of urban green space on residents’ health in Guangzhou, China. J Urban Planning Dev.

[16] Wu Y, Ye Z, Fang Y (2019). Spatial analysis of the effects of PM2. 5 on hypertension among the middle-aged and elderly people in China. Int J Environ Health Res.

[17] Leng H, Li S, Yan S, An X (2020). Exploring the relationship between green space in a neighbourhood and cardiovascular health in the winter city of China: a study using a health survey for harbin. Int J Environ Res Public Health.

[18] Curto A, Wellenius GA, Milà C, Sanchez M, Ranzani O, Marshall JD (2019). Ambient particulate air pollution and blood pressure in peri-urban India. Epidemiology.

[19] Xu H, Li H (2017). Total amount calculation and health benefit assessment of PM. 25 adsorbed by urban green space in Xuzhou City, China. Nat Environ Pollut Technol.

[20] Maas J, Verheij RA, Spreeuwenberg P, Groenewegen PP. Physical activity as a possible mechanism behind the relationship between green space and health: a multilevel analysis. BMC Public Health. 2008;8(1):206. 10.1186/1471-2458-8-206. 10.1186/1471-2458-8-206 PMC2438348 18544169

[21] Yang B-YQ, Qian Z, Howard SW, Vaughn MG, Fan S-J, Liu K-K (2018). Global association between ambient air pollution and blood pressure: a systematic review and meta-analysis. Environ Pollut.

[22] Li N, Chen G, Liu F, Mao S, Liu Y, Hou Y (2019). Associations of long-term exposure to ambient PM1 with hypertension and blood pressure in rural Chinese population: the Henan rural cohort study. Environ Int.

[23] Wu YY, Zirong Y, Fang Y (2019). Spatial analysis of the effects of PM2. 5 on hypertension among the middle-aged and elderly people in China. Int J Environ Health Res.

[24] Curto AW, Milà GA, Sanchez C, Ranzani M, Marshall O, Kulkarni JD (2019). Ambient particulate air pollution and blood pressure in peri-urban India. Epidemiology.

[25] Carey RM, Muntner P, Bosworth HB, Whelton PK (2018). Reprint of: prevention and control of hypertension: JACC health promotion series. J Am Coll Cardiol.

[26] Rahmati AR, Kiani B, Afshari A, Moghaddas E, Williams M, Shamsi S (2020). World-wide prevalence of *Anisakis larvae* in fish and its relationship to human allergic anisakiasis: a systematic review. Parasitol Res.

[27] Park S-Y, Kwak J-M, Seo E-W, Lee K-S (2016). Spatial analysis of the regional variation of hypertensive disease mortality and its socio-economic correlates in South Korea. Geospat Health.

[28] Kiani B, Bagheri N, Tara A, Hoseini B, Tabesh H, Tara M (2017). Revealed access to haemodialysis facilities in northeastern Iran: factors that matter in rural and urban areas. Geospat Health.

[29] Tohidinezhad F, Khorsand A, Zakavi SR, Rezvani R, Zarei-Ghanavati S, Abrishami M (2020). The burden and predisposing factors of non-communicable diseases in Mashhad University of Medical Sciences personnel: a prospective 15-year organizational cohort study protocol and baseline assessment. BMC Public Health.

[30] Kim D, Zhang Y, Lee CK (2018). Understanding needs and barriers to using geospatial tools for public health policymaking in China. Geospat Health.

[31] (MMPGSO) MMPaGSO. Statistics and information about Mashahd city parks at the end of 2020 Mashhad, Iran; 2020. https://parks.mashhad.ir//parameters/mashhad/modules/cdk/upload/content/file_manager/10864/list%20of%20parks.pdf. Accessed 20 Sept 2021.

[32] Shabanikiya H, Hashtarkhani S, Bergquist R, Bagheri N, VafaeiNejad R, Amiri-Gholanlou M (2020). Multiple-scale spatial analysis of paediatric, pedestrian road traffic injuries in a major city in North-Eastern Iran 2015–2019. BMC Public Health.

[33] World Health Organization (WHO). Cardiovascular diseases (CVDs) 2021. https://www.who.int/news-room/fact-sheets/detail/cardiovascular-diseases-(cvds). Accessed 20 Sept 2021.

[34] Brook RD, Rajagopalan S (2018). ACC/AHA/AAPA/ABC/ACPM/AGS/APhA/ASH/ASPC/NMA/PCNA guideline for the prevention, detection, evaluation, and management of high blood pressure in adults. A report of the American College of Cardiology/American Heart Association Task Force on Clinical Practice Guidelines. J Am Society Hypertension..

[35] Austin PC, Stryhn H, Leckie G, Merlo J (2018). Measures of clustering and heterogeneity in multilevel Poisson regression analyses of rates/count data. Stat Med.

[36] Hoseini B, Bagheri N, Kiani B, Azizi A, Tabesh H, Tara M (2018). Access to dialysis services: a systematic mapping review based on geographical information systems. Geospat Health.

[37] Kiani B, Raouf Rahmati A, Bergquist R, Moghaddas E (2020). Comparing spatio-temporal distribution of the most common human parasitic infections in Iran over two periods 2007 to 2012 and 2013 to 2018: a systematic quantitative literature review. Int J Health Plan Manag.

[38] Pishgar E, Mohammadi A, Bagheri N, Kiani B (2020). A spatio-temporal geodatabase of mortalities due to respiratory tract diseases in Tehran, Iran between 2008 and 2018: a data note. BMC Res Notes.

[39] Kiani B (2021). A geodatabase to measure the blood pressure level of individuals living in the city of Mashhad, Iran. Harvard Dataverse..

